# Supplementary material for: An online, open-access, cost comparison tool for presumptive tuberculosis testing using low complexity, automated, nucleic acid amplification tests (LCaNAAT) versus near point-of-care (NPOCs) molecular tests
Source: PLOS Glob Public Health. 2026 Nov 4;6(11):e0006559. doi: 10.1371/journal.pgph.0006559 (PMC13734499; doi:10.1371/journal.pgph.0006559)
Supplement: Supplementary file 1 [file pgph.0006559.s001.pdf]

S1 Table: Scenario 1 cost components of LCaNAAT & NPOC in Country A (in USD, 2025)

Component	Year 1	Year 2	Year 3		Component	Year 1	Year 2	Year 3
LCaNAAT					NPOC			
Total cartridge cost (incl. import & storage)	27,026,815	29,729,493	32,702,442		NPOC device cost (incl. import & storage)	1,687,795	-	-
Service & maintenance (Xpert)	3,201,600	3,201,600	3,201,600		NPOC test card cost (incl. import & storage)	11,742,785	8,970,182	9,867,200
Sample transport	1,552,687	1,707,956	1,878,751		LCaNAAT cartridge cost for DRTB evaluation (incl. import & storage)	1,788,734	1,967,607	2,164,368
					Service & maintenance (NPOC + DRTB)	354,968	354,968	354,968
					Sample transport	1,855,350	2,040,885	2,244,973
Total Cost (LCaNAAT)	31,781,102	34,639,049	37,782,793		Total Cost (NPOC)	17,429,633	13,333,643	14,631,510
