# Supplementary material for: An online, open-access, cost comparison tool for presumptive tuberculosis testing using low complexity, automated, nucleic acid amplification tests (LCaNAAT) versus near point-of-care (NPOCs) molecular tests
Source: PLOS Glob Public Health. 2026 Nov 4;6(11):e0006559. doi: 10.1371/journal.pgph.0006559 (PMC13734499; doi:10.1371/journal.pgph.0006559)
Supplement: Supplementary file 2 [file pgph.0006559.s002.pdf]

S2 Table: Scenario 2 cost components of LCaNAAT & NPOC in Country A (USD 2025)

Component	Year 1	Year 2	Year 3		Component	Year 1	Year 2	Year 3
LCaNAAT					NPOC			
Total cartridge cost (incl. import & storage)	31,219,444	32,571,171	33,981,426		NPOC device cost (incl. import & storage)	2,080,697	-	-
Device procurement & one-off import/storage	5,592,320	-	-		NPOC test card cost (incl. import & storage)	13,564,426	9,827,592	10,253,104
Service & maintenance (Xpert)	3,946,900	3,946,900	3,946,900		DRTB module cost (incl. import & storage)	2,066,218	2,155,680	2,249,016
Sample transport	1,793,553	1,871,210	1,952,229		Service & maintenance (NPOC + DRTB)	522,949	522,949	522,949
					Sample transport	1,246,391	1,300,357	1,356,659
Total Cost (LCaNAAT)	42,552,217	38,389,280	39,880,555		Total Cost (NPOC)	19,480,681	13,806,578	14,381,728
