# Supplementary material for: An online, open-access, cost comparison tool for presumptive tuberculosis testing using low complexity, automated, nucleic acid amplification tests (LCaNAAT) versus near point-of-care (NPOCs) molecular tests
Source: PLOS Glob Public Health. 2026 Nov 4;6(11):e0006559. doi: 10.1371/journal.pgph.0006559 (PMC13734499; doi:10.1371/journal.pgph.0006559)
Supplement: Supplementary file 3 [file pgph.0006559.s003.pdf]

S3 Table: Scenario 1 cost components of LCaNAAT & NPOC in Country B (USD 2025)

Component	Year 1	Year 2	Year 3		Component	Year 1	Year 2	Year 3
LCaNAAT					NPOC			
Total cartridge cost (incl. import & storage)	43,460,424	47,806,460	52,587,098		NPOC device cost (incl. import & storage)	4,000,752	-	-
Service & maintenance (Xpert)	5,637,600	5,637,600	5,637,600		NPOC test card cost (incl. import & storage)	18,882,966	14,424,486	15,866,932
Sample transport	7,419,050	8,160,954	8,977,048		DRTB module cost (incl. import & storage)	7,153,787	7,869,165	8,656,080
					Service & maintenance (NPOC + DRTB)	1,207,654	1,207,654	1,207,654
					Sample transport	6,915,201	7,606,721	8,367,391
Total Cost (LCaNAAT)	56,517,074	61,605,014	67,201,746		Total Cost (NPOC)	38,160,361	31,108,026	34,098,058
