# Supplementary material for: An online, open-access, cost comparison tool for presumptive tuberculosis testing using low complexity, automated, nucleic acid amplification tests (LCaNAAT) versus near point-of-care (NPOCs) molecular tests
Source: PLOS Glob Public Health. 2026 Nov 4;6(11):e0006559. doi: 10.1371/journal.pgph.0006559 (PMC13734499; doi:10.1371/journal.pgph.0006559)
Supplement: Supplementary file 4 [file pgph.0006559.s004.pdf]

S4 Table: Scenario 2 cost components of LCaNAAT & NPOC in Country B (USD 2025)

Component	Year 1	Year 2	Year 3		Component	Year 1	Year 2	Year 3
LCaNAAT					NPOC			
Total cartridge cost (incl. import & storage)	66,014,141	72,615,547	79,877,097		NPOC device cost (incl. import & storage)	8,079,708	-	-
Device procurement & one-off import/storage	56,685,200	-	-		NPOC test card cost (incl. import & storage)	28,682,251	21,910,050	24,101,054
Service & maintenance (Xpert)	11,385,400	11,385,400	11,385,400		DRTB module cost (incl. import & storage)	10,866,234	11,952,856	13,148,141
Sample transport	11,269,154	12,396,068	13,635,674		Service & maintenance (NPOC + DRTB)	2,429,391	2,429,391	2,429,391
					Sample transport	4,869,257	5,356,182	5,891,800
Total Cost (LCaNAAT)	145,353,895	96,397,015	104,898,171		Total Cost (NPOC)	54,926,841	41,648,479	45,570,385
