# Supplementary material for: An online, open-access, cost comparison tool for presumptive tuberculosis testing using low complexity, automated, nucleic acid amplification tests (LCaNAAT) versus near point-of-care (NPOCs) molecular tests
Source: PLOS Glob Public Health. 2026 Nov 4;6(11):e0006559. doi: 10.1371/journal.pgph.0006559 (PMC13734499; doi:10.1371/journal.pgph.0006559)
Supplement: Supplementary file 5 [file pgph.0006559.s005.docx]

This manuscript presents a useful and timely tool for estimating costs of TB diagnostic strategies and highlights the promise of NPOCs in terms of cost savings. The open-access and adaptable nature of the tool is a major strength. However, the analysis is limited to high-level cost comparisons which may constrain its relevance for policy decision-making. The manuscript would benefit from clearer positioning as an illustrative tool that can identify the savings that are possible from incorporating NPOC within a broader TB diagnostic network and stronger justification of assumptions.

**Major comments**

- The stated scope of the analysis would benefit from clearer alignment throughout the manuscript. While the discussion appropriately notes that the tool highlights the “promise” of NPOC diagnostics and is a high-level planning aid and does not replace detailed cost-effectiveness or budget impact analyses, earlier sections also position the tool as assessing the budget implications of integrating NPOC into existing diagnostic networks. These distinctions are important, as they imply different analytical perspectives and decision questions. The authors should more explicitly define the primary objective of the tool and ensure that the interpretation of results remains consistent with this scope.
- The authors are to be commended for developing a simple and accessible tool, and for highlighting the substantial potential of NPOC diagnostics as a game changer for improving access and expanding testing coverage, while maintaining acceptable performance. The primary objective of the analysis appears to be to illustrate the magnitude of potential cost savings, which are indeed considerable. However, the current analysis largely demonstrates that a lower-cost diagnostic technology results in lower programme costs under the assumptions specified (which favour NPOC), which is unsurprising and may limit its usefulness for decision-making.
  - In practice, TB diagnostic procurement decisions are not based on cost alone; while cost is a key driver, other factors such as diagnostic performance, platform multiplex functionality, existing infrastructure, and broader programme priorities also play an important role. A more policy-relevant question is therefore not whether NPOC is cheaper (which it clearly is), but under what conditions it may not be preferable. For example, in settings with a large decentralised LCaNAAT install base, or where differences in sensitivity and specificity may influence case detection, the relative value of NPOC may be less straightforward. Incorporating even simple performance parameters (e.g., sensitivity, specificity, and disease prevalence) would enable estimation of metrics such as cost per correct diagnosis and facilitate threshold or break-even analyses (e.g., identifying when a switch to NPOC may result in an unacceptable number of missed cases given the existing system). This would allow the authors to provide more actionable guidance, e.g. “NPOC is preferred unless X exceeds Y”—which is more aligned with real-world policy needs. As currently presented, the analysis does not fully address the potential trade-offs in diagnostic accuracy, case detection, or system functionality that may arise if NPOC were implemented at scale, even if these trade-offs are ultimately outweighed by the substantial gains in cost savings and expanded access.
- *Financial costs:* The analysis does not clearly distinguish between economic costs and financial (budget impact) costs. In many high-burden settings, LCaNAAT platforms such as GeneXpert are already installed, and capital costs are therefore sunk. Including these costs as avoidable may overstate the cost savings associated with NPOC implementation. I don’t think the authors have done this, but they need to clarify the costing perspective and be explicit about how capital costs are treated.
  - The treatment of capital costs suggests that the analysis is not conducted from an economic costing perspective, as equipment costs are included as capital expenditures within the 3-year horizon without annualisation or consideration of useful life (e.g. GeneXpert would have a longer lifespan than NPOC). If the intention is to conduct a budget impact analysis, the model should focus on the incremental costs of switching to an NPOC-based strategy, particularly given that much of the existing LCaNAAT infrastructure may already be in place and represent sunk costs (i.e. zero). This appears to be what is done but it is not clear – there is a “Device_procurement_if_any” variable in the documentation but cannot see how this is calculated. Would recommend being explicit about these assumptions.
  - I would also include a longer time horizon that may incorporate the longer working life of a LCaNAAT in a sensitivity analysis (as well as the shorter working life of NPOC devices).
- *Volumes. I*t was assumed that “each LCaNAAT module processes one test in 90~120 minutes and that each NPOC device processes one test in 30~40 minutes. This allows the NPOC device to complete two testing cycles in the time it takes one LCaNAAT/Xpert cycle, and based on operational efficiency, two NPOC test cards are estimated to match the daily testing capacity of one LCaNAAT module.”
  - The authors should ensure consistency between throughput assumptions and cost inputs (which are on a four-module LCaNAAT instrument).
  - I think leaving out HR is quite important. Eg what is the difference in hands on time between NPOC and LCaNAAT. Are we tying up human resources in high volume clinics when these tests could have been referred out to high volume lab equipment? This can be mentioned in the discussion.
  - The model appears to assume that current installed capacity has perfect utilisation as NPOC device volumes are calculated based off LCaNAAT modules. This is likely an oversimplification and should be clearly stated. In practice, utilisation can vary widely across settings (both over-capacity and chronic under-utilisation), and a more robust approach would be to derive capacity from explicit operational throughput assumptions (e.g. run time, working hours, and utilisation rates).
  - Assumptions regarding volumes and devices required need to be a lot clearer and justifications for any simplifications made need to be provided.

**Introduction**

- The introduction would benefit from clearer and more consistent definitions of the diagnostic technologies under consideration. The distinction between LCaNAAT and NPOC platforms is central to the analysis, yet the current terminology (LCaNAAT, mWRD, NPOC, NPOC-NAATs) is used somewhat interchangeably, which may be confusing and should be standardised early. It would also be helpful to briefly and systematically describe key characteristics of each technology class and differentiate for a reader that is not familiar with these terms (such as performance, cost, throughput, and typical use cases).
- In Line 72, state that the performance of NPOC is approaching the WHO TPP and earlier in Line 67 state that the NPOC has comparable accuracy to LCaNAAT. I think it would be helpful to explicitly state these results : sensitivity of x, y relative to WHO TPP. Perhaps this can form part of the technology description above. I think this is important for setting the stage that cost is important because performance is comparable.

**Methods**

- Cost inputs
  - What justification that costs will decrease by 30%: line 229: “and decreasing to USD 2.50 in subsequent years.”
  - Clarify whether cost inputs inputted on a per module basis for LCaNAAT (e.g. device cost of $19000/4)?

**Scenarios**

- The definition of the two scenarios is somewhat confusing and does not fully align with how I imagine current diagnostic scale-up decisions are typically made in practice. In Scenario 1, LCaNAAT is replaced by NPOC while microscopy appears to remain unchanged; however, I assume that the priority in many settings would be to first replace microscopy with molecular testing, rather than displacing existing LCaNAAT capacity. In contrast, Scenario 2 assumes that microscopy sites are replaced by both LCaNAAT and NPOC - does this mean NPOC is the initial test and LCaNAAT the referral? Perhaps clarify.
- Perhaps more intuitive and policy-relevant default scenarios might include: (i) current status quo with current testing volumes; (ii) replacing both microscopy and initial LCaNAAT testing with NPOC under current testing volumes; (iii) expanding overall testing coverage using NPOC. This will allow comparison with the status quo, the status quo volumes with NPOC, and then the expanding testing scenario.
- I couldn’t really see how to customize scenarios in the tool – especially which platforms are replaced by which device type in each scenario.

**Tool**

- What about countries with TrueNat capacity. Can you include TrueNat in the instructions (but may have implications for scaling – e.g. when do you need additional sample prep analyzers- not linear scaling, need to incorporate cost of analyzers). The analysis overstates the cost advantage of NPOC by using GeneXpert as the sole comparator, without considering alternative WHO-endorsed LCaNAAT platforms such as Truenat, which have different cost structures, modular scaling, and infrastructure requirements.
- “Number of microscopy sites without molecular testing” this question is actually asking for the number of microscopes (to determine capacity), not necessarily microscopy sites as there may be microscopy sites that also have LCaNAAT capacity or microscopy sites with more than one microscope?
- Useful output to include in the tool would be the number of devices that need procurement (e.g. X NPOC in Year 1). Also, the resultant spare capacity available on the LCaNAAT after the shift. This information would be useful to policy makers inputting budgets and determining spare capacity on their installed network and more closely align with the scope that this is a high-level planning tool.
- Perhaps include some basic error messages (e.g. if the number of presumptive tests with molecular testing is greater than the number of presumptive TB tests done in a year – then a error message pops up)
- Include the costs all in one section – e.g. put the assumptions used for additional costs down below the cost assumptions (in blue) and all testing target assumptions together

**Countries**

- The choice and presentation of the country scenarios could be strengthened. Firstly – why these two countries? They seem quite similar in diagnostic network structure but only differ on volume of tests? Are they illustrating something different? Provide justification for their selection – or was it merely because they had data available?
- Additionally, while presented as generic “Country A” and “Country B,” they are clearly based on Bangladesh and Indonesia, which creates some ambiguity; this should either be made explicit or the abstraction maintained consistently.
- To improve clarity, it would be helpful to present key characteristics (e.g., total tests, proportion of mWRD vs microscopy) and summarise parameters and assumptions for both countries side by side. Perhaps even a label would work – e.g. “high microscopy”

**Discussion**

- Discuss difference in NPOC vs LCaNAAT in discussion but think this should also form part of the methodology/introduction.
- More on limitations regarding staff burden
- The treatment of LCaNAAT capacity in the NPOC scenario is somewhat unrealistic. While capital costs could reasonably be treated as sunk in a budget impact analysis, service and maintenance costs are typically tied to the device or system as a whole and are not easily scaled down at the module level. In practice, it is unlikely that excess modules can be decommissioned or that service contracts can be reduced proportionally. The model also implicitly assumes that any unused LCaNAAT capacity can be absorbed by other programmes, which may not be the case—for example, a site may only require one module for TB, leaving the remaining modules underutilised with no alternative use. This “divisibility” issue is an important limitation and may lead to an underestimation of ongoing costs and an overstatement of the savings associated with NPOC. Note as a limitation.
- I think the discussion should mention how a diagnostic network optimisation analysis can assist with placement decisions and provide granularity on devices required that can inform a more detailed budget impact analysis.

**Minor comments**

- Clarity and readability: Some sentences are long and technical (especially abstract). Simplify for broader public health audience throughout:
  - E.g. Line 129: “but rather to estimate how different scales of sample testing expansion could affect overall program costs.” Rather “but rather to assess how varying levels of testing scale-up may impact overall programme costs.”
  - First sentence of introduction is hard to follow
  - Sentence on Line 66: Should the MiniDock MTB and Truenat MTB Ultima be included both as examples of NPOC (currently MiniDock MTB repeated mention and TrueNat only included at end of sentence). Later in Line 75 refer to the MiniDock MTB as “(e.g., MiniDock by Pluslife)” – be consistent.
  - Consistency with abbreviations – e.g. near POC in Line 73 to NPOC.
- Tables: Large cost tables (e.g., Tables 3 & 4): Hard to interpret quickly. Suggest adding per test costs or cost per case detected.
- Missing links: App and code are referenced but not clearly linked in manuscript. Ensure accessible URLs
- Is the correct word not “microscopy sites” instead of “microscopic sites”?
- Very little justification or references provided for assumptions.
  - (e.g. line 212 assumed all tests start at 5.6m in year 1)
  - E.g. line 192: “We thought that when NPOC is rolled out, the number of samples needed to be transferred to LCaNAAT site for primary testing will go down to 5% in Scenario 1.”
- This phrase undermines credibility and reads as circular and unscientific. “Additionally, storage and distribution costs were conservatively estimated at 2% based on our assumption.” Please check for more of these and word better, for example: “Additionally, storage and distribution costs were assumed to be 2% of baseline costs, based on simplifying assumptions in the absence of empirical data.”
