# Supplementary material for: An online, open-access, cost comparison tool for presumptive tuberculosis testing using low complexity, automated, nucleic acid amplification tests (LCaNAAT) versus near point-of-care (NPOCs) molecular tests
Source: PLOS Glob Public Health. 2026 Nov 4;6(11):e0006559. doi: 10.1371/journal.pgph.0006559 (PMC13734499; doi:10.1371/journal.pgph.0006559)
Supplement: Supplementary file 6 [file pgph.0006559.s006.docx]

**Manuscript number**	PGPH-D-26-00841

**Title:** An online, open-access, cost comparison tool for presumptive tuberculosis testing

using low complexity, automated, nucleic acid amplification tests (LCaNAAT) versus

near point-of-care (NPOCs) molecular tests

**Overall comments:** This manuscript focused on an important area of development of automated cost calculation software or tool (flexible cost calculator) especially relevant for health economics. I hope this tool will help decision makers understand the financial implications of expanding the newer diagnostic tools for TB detection with optimising resource use and accelerating TB case detection under the National TB Control Programme. They also provided results of two hypothetical scenarios for Bangladesh and Indonesia. Overall, this research addresses an important topic and well written manuscript, I recommend for publication subject to minor revisions. However, the following points should be emphasised.

**Specific comments**

**Features of the tool**

- Can we call this tool as a cost estimation software for TB diagnosis?
- Whether this tool can provide cost estimates for different TB diagnostic algorithms? Since TB is not diagnosed by a single tool; it is a combination of tools following the diagnostic algorithm.
- It is stated that the purpose of this tool is not to conduct cost-effectiveness analyses. However, it would be useful to clarify whether the tool can generate any cost-effectiveness outputs. If not, can it be considered suitable for cost-minimisation analysis. Otherwise, costing alone, when assessed independently, may not provide full economic evaluation.
- Is it possible to add or modify the input parameters in the model or is it fixed?
- Is there any uncertainty analysis included in this tool? To find out how robust are the results.
- Whether a user manual or in-built guidance is available within the tool to support users in understanding and effectively utilizing its features
- Whether the auto generated graphs are editable
- It would be useful to clarify whether the tool has an automated report generation feature.

**Scenario analysis**

- The authors have currently used two scenarios. It would be useful to explore whether additional scenarios could be included. If so, is there a maximum number of scenarios that the tool can accommodate. This can be added in the manuscript.
- When calculating cumulative costs over the years, whether discount or inflation is taken into consideration
- This hypothetical model appears to overestimate cost savings, reporting 56.4% in country-A for Scenario-1 based on current testing trend and 60.5% in country –B for Scenario-2 based on ambitious testing goals. This may require further justification or sensitivity analysis to support these estimates.
- It was mentioned that the results highlight the potential of cheaper molecular diagnostics to significantly improve access to mWRDs. It needs clarity.

**Other comments**

- It is unclear whether the manuscript aims to highlight the innovation of the tool itself or the results generated by the tool. This lack of clarity appears to dilute the main focus of the study**.**
- Are there any limitations for the tool and scenario analysis?
- Conclusion is not highlighting the tool
- Tables beginning with Table number 10.
- All tables require professional editing to improve formatting.
- The first reference seems incomplete.

Overall, authors made a good effort, but the current version needs a revision.
