# Supplementary material for: An online, open-access, cost comparison tool for presumptive tuberculosis testing using low complexity, automated, nucleic acid amplification tests (LCaNAAT) versus near point-of-care (NPOCs) molecular tests
Source: PLOS Glob Public Health. 2026 Nov 4;6(11):e0006559. doi: 10.1371/journal.pgph.0006559 (PMC13734499; doi:10.1371/journal.pgph.0006559)
Supplement: Supplementary file 7 [file pgph.0006559.s007.docx]

April 29, 2026

Priya Rajendran, PhD
Academic Editor
PLOS Global Public Health

Dear Dr.Rajendran,

Thank you for your careful review of our manuscript, “An online, open-access cost comparison tool for presumptive tuberculosis testing using low-complexity automated nucleic acid amplification tests (LCaNAAT) versus near point-of-care (NPOC) molecular tests”(PGPH-D-26-00841).

We greatly appreciate the thoughtful and constructive comments provided. We have addressed all 58 comments in detail, and a comprehensive, point-by-point response is enclosed in the accompanying table.

We would also like to respectfully clarify that the manuscript was submitted under the *Methods, Software, Databases, and Tools* category. Accordingly, the primary objective of the paper is to present and describe the tool’s development and functionality, rather than to conduct a comprehensive economic evaluation, such as a budget impact analysis.

While we value all reviewer feedback, we note that some comments from Reviewer One appear to pertain more specifically to budget impact analysis. In our responses, we have clarified the manuscript's scope and provided justification for omitting those elements.

Please do not hesitate to let us know if any additional information or clarification is required. We would be happy to provide further details.

Thank you again for your time and consideration. We look forward to your response.

Yours sincerely,

Pushpita

Comments from Reviewer 1

|  | Reviewer comments | Response |
| --- | --- | --- |
| 1. | This manuscript presents a useful and timely tool for estimating costs of TB diagnostic strategies and highlights the promise of NPOCs in terms of cost savings. The open-access and adaptable nature of the tool is a major strength. However, the analysis is limited to high-level cost comparisons which may constrain its relevance for policy decision-making. The manuscript would benefit from clearer positioning as an illustrative tool that can identify the savings that are possible from incorporating NPOC within a broader TB diagnostic network and stronger justification of assumptions. | Thank you for your thoughtful and constructive feedback. We have revised the manuscript to clarify its scope and positioning. The primary objective of this work is to describe the development and functionality of the tool rather than to conduct a full economic evaluation or prescribe specific policy strategies.  The tool is intended as a practical, user-friendly calculator that enables stakeholders to estimate and compare the costs of TB diagnostic testing using LCaNAAT and NPOC approaches under varying testing volumes. It was developed in close consultation with two national TB programs and has been iteratively refined through testing in multiple low-resource settings. Feedback from these stakeholders has been incorporated to ensure its relevance and usability for policymakers.  We have also strengthened the manuscript to better emphasize that the tool serves an illustrative purpose—helping users explore potential cost implications and identify where savings may be realized by incorporating NPOC within broader diagnostic networks. Additionally, we have provided clearer justification of key assumptions and highlighted the tool’s limitations, particularly its focus on high-level cost comparisons rather than comprehensive economic or policy analysis. |
|  | **Major comments** |  |
| 2. | The stated scope of the analysis would benefit from clearer alignment throughout the manuscript. While the discussion appropriately notes that the tool highlights the “promise” of NPOC diagnostics and is a high-level planning aid and does not replace detailed cost-effectiveness or budget impact analyses, earlier sections also position the tool as assessing the budget implications of integrating NPOC into existing diagnostic networks. These distinctions are important, as they imply different analytical perspectives and decision questions. The authors should more explicitly define the primary objective of the tool and ensure that the interpretation of results remains consistent with this scope. | Thank you for this detailed and thoughtful set of comments. We have revised the manuscript to improve conceptual clarity, strengthen alignment across sections, and better reflect the intended scope and appropriate interpretation of the tool.  First, we now more explicitly define the primary objective of the tool early in the manuscript (Background and Methods). The tool is positioned as a high-level budget planning and procurement support instrument designed to estimate the financial implications of scaling and integrating NPOC diagnostics under different programmatic scenarios. We clearly state (lines 99–111) that the purpose of the tool is to describe and operationalize cost estimation, not to conduct formal cost-effectiveness or budget impact analyses. This distinction is now consistently maintained throughout the Results and Discussion, ensuring that the interpretation of findings aligns with the tool’s intended use.  Second, we have strengthened the framing to emphasize that the tool is designed to support scenario-based, order-of-magnitude cost comparisons rather than to determine optimal strategies. It was intentionally developed as a simple, transparent, and adaptable calculator in close consultation with national TB program stakeholders across multiple low-resource settings. These users prioritized ease of use and rapid estimation to inform planning and procurement discussions, particularly in contexts where more complex economic evaluations may not be feasible.  We agree with the reviewer that real-world decision-making extends beyond cost considerations. Accordingly, we have expanded the Discussion to more explicitly acknowledge that factors such as diagnostic performance, existing infrastructure (including installed LCaNAAT capacity), multiplexing functionality, and broader programmatic priorities are critical in shaping procurement decisions. We also clarify that the tool allows users to adapt key cost inputs—such as installation costs—to reflect local realities (e.g., existing infrastructure), reinforcing that it is intended to complement, not replace, more comprehensive analyses.  We appreciate the suggestion to incorporate diagnostic performance parameters and threshold analyses (e.g., cost per correct diagnosis, break-even conditions). While these additions would enhance policy relevance, they would also substantially increase model complexity and data requirements, which runs counter to the tool’s core objective of accessibility and usability. Nevertheless, we recognize the value of this approach and have added text in the Discussion identifying it as an important direction for future development.  Finally, we clarify that while the tool incorporates country-specific epidemiological inputs (such as testing volumes and positivity rates) to better contextualize cost estimates, it does not model diagnostic accuracy, case detection, or downstream health outcomes. The tool should therefore be interpreted as supporting financial planning conditional on a chosen diagnostic strategy, rather than as a framework for determining the comparative effectiveness or overall value of alternative approaches.  These revisions aim to ensure that the manuscript presents a coherent and appropriately scoped contribution, while acknowledging both the strengths and limitations of the tool in informing policy decisions. |
| 3. | The authors are to be commended for developing a simple and accessible tool, and for highlighting the substantial potential of NPOC diagnostics as a game changer for improving access and expanding testing coverage, while maintaining acceptable performance. The primary objective of the analysis appears to be to illustrate the magnitude of potential cost savings, which are indeed considerable. However, the current analysis largely demonstrates that a lower-cost diagnostic technology results in lower programme costs under the assumptions specified (which favour NPOC), which is unsurprising and may limit its usefulness for decision-making. In practice, TB diagnostic procurement decisions are not based on cost alone; while cost is a key driver, other factors such as diagnostic performance, platform multiplex functionality, existing infrastructure, and broader programme priorities also play an important role. A more policy-relevant question is therefore not whether NPOC is cheaper (which it clearly is), but under what conditions it may not be preferable. For example, in settings with a large decentralised LCaNAAT install base, or where differences in sensitivity and specificity may influence case detection, the relative value of NPOC may be less straightforward. Incorporating even simple performance parameters (e.g., sensitivity, specificity, and disease prevalence) would enable estimation of metrics such as cost per correct diagnosis and facilitate threshold or break-even analyses (e.g., identifying when a switch to NPOC may result in an unacceptable number of missed cases given the existing system). This would allow the authors to provide more actionable guidance, e.g. “NPOC is preferred unless X exceeds Y”—which is more aligned with real-world policy needs. As currently presented, the analysis does not fully address the potential trade-offs in diagnostic accuracy, case detection, or system functionality that may arise if NPOC were implemented at scale, even if these trade-offs are ultimately outweighed by the substantial gains in cost savings and expanded access. |  |
|  |  |  |
| 4. | *Financial costs:* The analysis does not clearly distinguish between economic costs and financial (budget impact) costs. In many high-burden settings, LCaNAAT platforms such as GeneXpert are already installed, and capital costs are therefore sunk. Including these costs as avoidable may overstate the cost savings associated with NPOC implementation. I don’t think the authors have done this, but they need to clarify the costing perspective and be explicit about how capital costs are treated. | Thank you for this important comment. We have clarified the costing perspective in the revised manuscript to avoid ambiguity.  This study does not adopt a formal economic costing framework; rather, the tool is designed as a financial planning and budgeting support instrument. Accordingly, a full micro-costing approach was not undertaken.  In line with this perspective:  Existing LCaNAAT infrastructure is treated as sunk, and therefore no additional procurement or installation costs are applied to LCaNAAT platforms already in place.  In contrast, NPOC-related costs include procurement and installation, reflecting the incremental financial investment required for adoption and scale-up.  We have also clarified that certain cost components—such as building infrastructure, overheads, and electricity—are not included, as the tool is intended to provide simplified, order-of-magnitude estimates aligned with program-level budgeting needs.  The purpose of the tool is to support practical decision-making by allowing users to assess whether introducing NPOC for primary testing is financially feasible under different testing volumes. While LCaNAAT platforms may already be installed in many settings, their per-test and maintenance costs remain relevant for ongoing budgeting decisions, particularly when testing volumes increase.  To reflect real-world conditions, we included a setting with an extensive existing LCaNAAT network (e.g., Bangladesh). Even under these conditions—where capital costs are largely sunk—the model demonstrates that NPOC-based testing may still offer cost savings at higher testing volumes. We have clarified this interpretation in the manuscript to ensure it is not overstated. |
| 5. | The treatment of capital costs suggests that the analysis is not conducted from an economic costing perspective, as equipment costs are included as capital expenditures within the 3-year horizon without annualisation or consideration of useful life (e.g. GeneXpert would have a longer lifespan than NPOC). If the intention is to conduct a budget impact analysis, the model should focus on the incremental costs of switching to an NPOC-based strategy, particularly given that much of the existing LCaNAAT infrastructure may already be in place and represent sunk costs (i.e. zero). This appears to be what is done but it is not clear – there is a “Device_procurement_if_any” variable in the documentation but cannot see how this is calculated. Would recommend being explicit about these assumptions. | We clarify that the tool is neither an economic evaluation nor a formal budget impact model. Rather, it is a simplified decision-support calculator designed to answer “what-if” questions related to the financial implications of introducing NPOC for primary testing and reflex testing under different programmatic scenarios. Users can adjust procurement inputs depending on whether incremental scale-up or full replacement is being considered.  The tool is intended to reflect how procurement decisions are typically operationalized within real-world programmatic and funding contexts, including standard procurement cycles such as those supported by the Global Fund, where initial investment decisions are often made upfront.  Importantly, the tool is designed to support countries in planning and budgeting for the achievement of UNGA targets by providing a simplified framework to explore how different diagnostic scale-up strategies may affect overall program costs. In this context, potential savings from NPOC adoption may allow reallocation of resources to other priority areas within national TB programs.  All model assumptions are transparently documented in Table 1. |
| 6. | I would also include a longer time horizon that may incorporate the longer working life of a LCaNAAT in a sensitivity analysis (as well as the shorter working life of NPOC devices). | We thank the reviewer for this important suggestion. The selected time horizon was chosen to align with typical programmatic and procurement planning cycles, particularly those used by major funding mechanisms such as the Global Fund. This reflects how diagnostic investments are commonly assessed in practice, where procurement decisions are made within fixed funding and implementation cycles rather than over full equipment lifespans. |
| 7. | *Volumes. I*t was assumed that “each LCaNAAT module processes one test in 90~120 minutes and that each NPOC device processes one test in 30~40 minutes. This allows the NPOC device to complete two testing cycles in the time it takes one LCaNAAT/Xpert cycle, and based on operational efficiency, two NPOC test cards are estimated to match the daily testing capacity of one LCaNAAT module.” The authors should ensure consistency between throughput assumptions and cost inputs (which are on a four-module LCaNAAT instrument). | All calculations are conducted at the module level for LCaNAAT platforms, rather than at the device level. Accordingly, cost inputs for LCaNAAT are based on a four-module system, and all throughput assumptions are standardized per module to ensure consistency between capacity and costing. |
| 8. | I think leaving out HR is quite important. Eg what is the difference in hands on time between NPOC and LCaNAAT. Are we tying up human resources in high volume clinics when these tests could have been referred out to high volume lab equipment? This can be mentioned in the discussion. | We thank the reviewer for raising this important point regarding human resource implications. We agree that differences in hands-on time and workflow between NPOC and LCaNAAT platforms may have operational relevance, particularly in high-volume settings where staffing constraints and task allocation can influence service delivery.  However, the current tool is designed specifically as a financial planning calculator focused on procurement and recurrent testing costs, rather than a full health systems or operational efficiency model. Human resource costs and workflow implications are therefore not explicitly included, as these are typically managed separately within national program planning and are not directly bundled with diagnostic procurement decisions.  We have now added a statement in the Discussion acknowledging that differences in staff time and operational workflow between platforms may influence implementation considerations and should be taken into account alongside financial outputs when interpreting results. |
| 9. | The model appears to assume that current installed capacity has perfect utilisation as NPOC device volumes are calculated based off LCaNAAT modules. This is likely an oversimplification and should be clearly stated. In practice, utilisation can vary widely across settings (both over-capacity and chronic under-utilisation), and a more robust approach would be to derive capacity from explicit operational throughput assumptions (e.g. run time, working hours, and utilisation rates). | We acknowledge that diagnostic capacity utilization can vary substantially across settings, including both under- and over-utilization of existing LCaNAAT platforms.  In the current tool, we adopt a simplified approach in which NPOC volumes are derived from LCaNAAT module capacity assumptions. This was done intentionally to maintain model simplicity and usability, and to provide a standardized basis for scenario comparison across settings.  We would like to clarify that this is not intended to reflect actual utilization efficiency or to assess performance of existing national systems. Rather, it provides a consistent reference framework for estimating relative costs under different deployment configurations.  We also note that in practice, countries may adjust NPOC deployment based on facility-level testing volumes, with higher numbers of devices in high-throughput sites and fewer in low-volume settings. |
| 10. | Assumptions regarding volumes and devices required need to be a lot clearer and justifications for any simplifications made need to be provided. | Please refer to table 1, all assumptions are listed there |
|  | **Introduction** |  |
| 11. | The introduction would benefit from clearer and more consistent definitions of the diagnostic technologies under consideration. The distinction between LCaNAAT and NPOC platforms is central to the analysis, yet the current terminology (LCaNAAT, mWRD, NPOC, NPOC-NAATs) is used somewhat interchangeably, which may be confusing and should be standardised early. It would also be helpful to briefly and systematically describe key characteristics of each technology class and differentiate for a reader that is not familiar with these terms (such as performance, cost, throughput, and typical use cases). | Definitions have been added , revised as follow:  Fewer than half of tuberculosis (TB) diagnostic units in 23 of 30 high-burden countries had access to World Health Organization (WHO)-recommended molecular rapid diagnostic tests (mWRDs) - a broad class of WHO-endorsed nucleic acid amplification tests (NAATs) capable of simultaneous TB detection and drug susceptibility testing (DST) — and these were used as the initial test in fewer than half of newly diagnosed TB cases in 2024[1]. The most widely deployed platforms in this class are the Xpert MTB/RIF Ultra (Cepheid) and Truenat (Molbio), both low-complexity automated NAATs (LCaNAATs). To date, high maintenance costs, unsustainable donor-reliant funding models, and limited diagnostic access at the community level have obstructed the scale-up of mWRDs. While patients and providers consistently value fast, accurate, and affordable testing, TB programs often fail to meet these expectations [2]. Moreover, the sharp reduction in funding by the United States and other Group of Seven (G7) nations has introduced new uncertainty, threatening the End TB Strategy and the Global Plan 2023–2030 [3]. Even modest donor cutbacks could result in millions of additional TB cases and hundreds of thousands of preventable deaths by 2035 [4]. In this uncertain context, high burden countries will have to do more with less funding, invest more domestic resources, and be more self-reliant [5].  Thankfully, a new emerging class of simplified, portable molecular tests — near-point-of-care NAATs (NPOC-NAATs), hereafter referred to as NPOC platforms, (e.g., MiniDock MTB Test (MiniDock MTB))- have been developed for decentralized deployment at peripheral health facilities and community settings. Unlike LCaNAATs, NPOC platforms require minimal infrastructure, can accommodate non-sputum specimens such as tongue swabs, and are substantially lower in cost. Multicounty evaluations have shown that the NPOC achieves a sensitivity of 91.1% (95% CI: 82.1–95.9) and specificity exceeding 98% on sputum swabs [6], with WHO-pooled data across studies reporting a summary sensitivity of 85.4% (95% CI: 82.2–88.1) and specificity of 97.6% (95% CI: 96.9–98.1) on sputum. These estimates meet or approach the WHO Target Product Profile (TPP) thresholds of ≥90% sensitivity and ≥98% specificity for near-POC TB tests on sputum, and substantially outperform smear microscopy. Importantly, with sputum swab-based testing, sensitivity for NPOC differed by only −3.0% (95% CI: −8.6, 2.6) compared to sputum Xpert Ultra, with specificity exceeding 98% [6] — a margin that is not statistically significant and supports the clinical equivalence of the two platforms in the populations studied. |
| 12. | In Line 72, state that the performance of NPOC is approaching the WHO TPP and earlier in Line 67 state that the NPOC has comparable accuracy to LCaNAAT. I think it would be helpful to explicitly state these results : sensitivity of x, y relative to WHO TPP. Perhaps this can form part of the technology description above. I think this is important for setting the stage that cost is important because performance is comparable. | Defitions have been added , revised as follow:  Fewer than half of tuberculosis (TB) diagnostic units in 23 of 30 high-burden countries had access to World Health Organization (WHO)-recommended molecular rapid diagnostic tests (mWRDs) - a broad class of WHO-endorsed nucleic acid amplification tests (NAATs) capable of simultaneous TB detection and drug susceptibility testing (DST) — and these were used as the initial test in fewer than half of newly diagnosed TB cases in 2024[1]. The most widely deployed platforms in this class are the Xpert MTB/RIF Ultra (Cepheid) and Truenat (Molbio), both low-complexity automated NAATs (LCaNAATs). To date, high maintenance costs, unsustainable donor-reliant funding models, and limited diagnostic access at the community level have obstructed the scale-up of mWRDs. While patients and providers consistently value fast, accurate, and affordable testing, TB programs often fail to meet these expectations [2]. Moreover, the sharp reduction in funding by the United States and other Group of Seven (G7) nations has introduced new uncertainty, threatening the End TB Strategy and the Global Plan 2023–2030 [3]. Even modest donor cutbacks could result in millions of additional TB cases and hundreds of thousands of preventable deaths by 2035 [4]. In this uncertain context, high burden countries will have to do more with less funding, invest more domestic resources, and be more self-reliant [5].  Thankfully, a new emerging class of simplified, portable molecular tests — near-point-of-care NAATs (NPOC-NAATs), hereafter referred to as NPOC platforms, (e.g., MiniDock MTB Test (MiniDock MTB))- have been developed for decentralized deployment at peripheral health facilities and community settings. Unlike LCaNAATs, NPOC platforms require minimal infrastructure, can accommodate non-sputum specimens such as tongue swabs, and are substantially lower in cost. Multicounty evaluations have shown that the NPOC achieves a sensitivity of 91.1% (95% CI: 82.1–95.9) and specificity exceeding 98% on sputum swabs [6], with WHO-pooled data across studies reporting a summary sensitivity of 85.4% (95% CI: 82.2–88.1) and specificity of 97.6% (95% CI: 96.9–98.1) on sputum. These estimates meet or approach the WHO Target Product Profile (TPP) thresholds of ≥90% sensitivity and ≥98% specificity for near-POC TB tests on sputum, and substantially outperform smear microscopy. Importantly, with sputum swab-based testing, sensitivity for NPOC differed by only −3.0% (95% CI: −8.6, 2.6) compared to sputum Xpert Ultra, with specificity exceeding 98% [6] — a margin that is not statistically significant and supports the clinical equivalence of the two platforms in the populations studied. |
|  | **Methods** |  |
|  | - Cost inputs |  |
| 13. | What justification that costs will decrease by 30%: line 229: “and decreasing to USD 2.50 in subsequent years.” | Justification was provided at the table footnote. it was assumed that the test cost would decrease as test volume increased. |
| 14. | Clarify whether cost inputs inputted on a per module basis for LCaNAAT (e.g. device cost of $19000/4)? | Revised as below: LCaNAAT cost parameters are pre-populated using the Global Drug Facility (GDF) pricing catalog but may be customized by users to reflect negotiated rates, national tender agreements, or manufacturer-specific terms. In this model, for the device’s price and the installation cost, we have used the price of a four-module GeneXpert. Service costs were estimated by dividing the total device warranty by the module count, using a conservative baseline of USD 725. The baseline of USD 725 per module represents the estimated cost of a standard extended warranty covering basic parts and module replacement, providing a conservative lower bound relative to premium GDF Service Level Agreements like Warranty. |
|  | **Scenarios** |  |
| 15. | The definition of the two scenarios is somewhat confusing and does not fully align with how I imagine current diagnostic scale-up decisions are typically made in practice. In Scenario 1, LCaNAAT is replaced by NPOC while microscopy appears to remain unchanged; however, I assume that the priority in many settings would be to first replace microscopy with molecular testing, rather than displacing existing LCaNAAT capacity. In contrast, Scenario 2 assumes that microscopy sites are replaced by both LCaNAAT and NPOC - does this mean NPOC is the initial test and LCaNAAT the referral? Perhaps clarify. | We respectfully clarify that the scenarios are described in detail in lines 211–236 of the manuscript. The goal of this paper is not to propose or evaluate any diagnostic replacement algorithm, but rather to provide national TB programs with a practical tool to assess whether transitioning to NPOC platforms can help them achieve their mWRD testing targets within existing budget constraints. Scenario 1 does not propose replacing microscopy; rather, it addresses a question of direct programmatic relevance to countries that already have established LCaNAAT networks — namely, whether switching to NPOC for primary molecular testing would yield cost savings while maintaining existing testing volumes. This is precisely the question the reviewer raises earlier in their comments, and we are confident that the scenario directly addresses it. Scenario 2, on the other hand, addresses a scale-up question: for countries seeking to expand testing by transitioning microscopy sites to molecular testing, what would be the comparative cost of doing so using LCaNAAT alone versus NPOC as the primary test with LCaNAAT retained for reflex testing? These are distinct, policy-relevant questions that reflect the real programmatic decisions currently facing high-burden countries, and we maintain that the two scenarios together provide a coherent and useful analytical framework |
| 16. | Perhaps more intuitive and policy-relevant default scenarios might include: (i) current status quo with current testing volumes; (ii) replacing both microscopy and initial LCaNAAT testing with NPOC under current testing volumes; (iii) expanding overall testing coverage using NPOC. This will allow comparison with the status quo, the status quo volumes with NPOC, and then the expanding testing scenario. | We appreciate the reviewer's suggestion; however, we respectfully submit that all three proposed scenarios are already captured within the analytical framework presented in this paper. Specifically, the status quo baseline, the substitution of existing testing platforms with NPOC at current volumes, and the scale-up of testing coverage using NPOC are each addressed — either directly through the two scenarios or through the comparative discussion presented in the Discussion section, where we explicitly draw these comparisons to contextualize the tool's outputs. We therefore do not consider restructuring the scenarios to be necessary and maintain that the current framework adequately covers the analytical ground the reviewer has identified. |
|  | I couldn’t really see how to customize scenarios in the tool – especially which platforms are replaced by which device type in each scenario. | The scenario customization functionality is built into the tool and is reflected directly in the results table, where the differential outputs across platform configurations are clearly displayed for comparison. We respectfully suggest that a closer review of the results table within the tool will make the platform substitution logic apparent. |
|  | **Tool** |  |
| 17. | What about countries with TrueNat capacity. Can you include TrueNat in the instructions (but may have implications for scaling – e.g. when do you need additional sample prep analyzers- not linear scaling, need to incorporate cost of analyzers). The analysis overstates the cost advantage of NPOC by using GeneXpert as the sole comparator, without considering alternative WHO-endorsed LCaNAAT platforms such as Truenat, which have different cost structures, modular scaling, and infrastructure requirements. | Thank you for your suggestion. Please note that the tool is designed to be user-friendly and adaptable, allowing countries to fully customize costs—such as specific warranty and service charges—to directly address the "different cost structures" mentioned in your comment. This flexibility is supported by the clear cost equivalence between platforms; device prices are comparable ($18,000 vs. $19,000) with a matching $7.50 cost-per-test. Furthermore, as the vast majority of countries already maintain an extensive, established GeneXpert network, utilizing it as the primary baseline reflects the most practical scaling path. However, should a country wish to evaluate TrueNat within the tool, they can simply input the relevant cost data to generate a customized estimation. |
| 18. | “Number of microscopy sites without molecular testing” this question is actually asking for the number of microscopes (to determine capacity), not necessarily microscopy sites as there may be microscopy sites that also have LCaNAAT capacity or microscopy sites with more than one microscope? | As explained in the instruction it refers to the number of microscope sites (not mWRD sites) that you plan to replace with NPOC in Scenario 2.  Please refer to the instruction |
| 19. | Useful output to include in the tool would be the number of devices that need procurement (e.g. X NPOC in Year 1). Also, the resultant spare capacity available on the LCaNAAT after the shift. This information would be useful to policy makers inputting budgets and determining spare capacity on their installed network and more closely align with the scope that this is a high-level planning tool. | Addressed. Here is a screenshot  |
| 20. | Perhaps include some basic error messages (e.g. if the number of presumptive tests with molecular testing is greater than the number of presumptive TB tests done in a year – then a error message pops up) | The tool wants to give flexibility to the users to play around the numbers, error messages can create further confusions. |
| 21. | Include the costs all in one section – e.g. put the assumptions used for additional costs down below the cost assumptions (in blue) and all testing target assumptions together | The cost costs shared by both LCaNAAT and NPOC have been grouped together. Separating it out can create confusion. |
|  | **Countries** |  |
| 22. | The choice and presentation of the country scenarios could be strengthened. Firstly – why these two countries? They seem quite similar in diagnostic network structure but only differ on volume of tests? Are they illustrating something different? Provide justification for their selection – or was it merely because they had data available? | Revised as follow:  We utilized publicly available data and personal communications to model two hypothetical scenarios, Country A and Country B, which were selected to represent contrasting logistical and economic archetypes. Both countries expressed a specific interest in participating in this study, providing a unique opportunity to validate the model using real-world data from diverse settings:  Country A was chosen to represent a compact, high-density geography where decentralization is logistically streamlined (e.g., Bangladesh). It also models a favorable fiscal environment where diagnostic tools are exempt from customs duties.  Country B was selected to represent a vast, geographically fragmented setting where decentralization faces significant logistical hurdles (e.g. Indonesia). This scenario also accounts for a high-cost import environment, allowing the tool to demonstrate how trade barriers impact diagnostic scaling.  By using these two distinct profiles, the analysis illustrates how the benefits of decentralization vary across different national landscapes without relying on a single, uniform scenario. |
| 23. | Additionally, while presented as generic “Country A” and “Country B,” they are clearly based on Bangladesh and Indonesia, which creates some ambiguity; this should either be made explicit or the abstraction maintained consistently. | See above |
| 24. | To improve clarity, it would be helpful to present key characteristics (e.g., total tests, proportion of mWRD vs microscopy) and summarise parameters and assumptions for both countries side by side. Perhaps even a label would work – e.g. “high microscopy” | Thank you for this helpful suggestion. While a side-by-side presentation could facilitate direct comparison, we have chosen a sequential structure to maintain a clear narrative flow—presenting Country A in full before moving to Country B. This approach helps avoid fragmentation and allows readers to better interpret each setting independently. We have, however, made sure that key characteristics and assumptions are explicitly summarised within each section to support clarity. |
|  | **Discussion** |  |
| 25. | Discuss difference in NPOC vs LCaNAAT in discussion but think this should also form part of the methodology/introduction. | Added in the introduction |
| 26. | More on limitations regarding staff burden | Explained earlier |
| 27. | The treatment of LCaNAAT capacity in the NPOC scenario is somewhat unrealistic. While capital costs could reasonably be treated as sunk in a budget impact analysis, service and maintenance costs are typically tied to the device or system as a whole and are not easily scaled down at the module level. In practice, it is unlikely that excess modules can be decommissioned or that service contracts can be reduced proportionally. The model also implicitly assumes that any unused LCaNAAT capacity can be absorbed by other programmes, which may not be the case—for example, a site may only require one module for TB, leaving the remaining modules underutilised with no alternative use. This “divisibility” issue is an important limitation and may lead to an underestimation of ongoing costs and an overstatement of the savings associated with NPOC. Note as a limitation. | We agree that in real-world settings, service and maintenance costs are often structured at the system level and may not scale proportionally with reductions in testing volumes or module utilization.  In the model, we assume that LCaNAAT platforms remain in use for reflex testing and other diagnostic functions even when NPOC is introduced for primary testing. As a result, LCaNAAT capacity is not assumed to be decommissioned, but rather partially re-purposed within the diagnostic network. This reflects common programmatic practice where molecular platforms are used across multiple testing algorithms (e.g., first-line testing, reflex testing, and drug susceptibility testing).  We acknowledge, however, that the degree to which excess capacity can be reallocated varies across settings. In some contexts, remaining capacity may indeed be underutilized if alternative diagnostic demand is limited. This represents a valid limitation of the model’s simplifying assumption regarding capacity divisibility and may influence the magnitude of estimated savings.  . |
| 28. | I think the discussion should mention how a diagnostic network optimisation analysis can assist with placement decisions and provide granularity on devices required that can inform a more detailed budget impact analysis. | Added , revised as follow: A key additional consideration is that this analysis is a simplified, high-level costing tool and does not capture spatial or facility-level optimization of diagnostic networks. In practice, diagnostic network optimization analyses can provide important complementary insights by identifying optimal device placement, estimating realistic utilization rates, and determining required capacity across health system tiers. Such approaches could further refine investment planning and inform more detailed budget impact analyses by linking cost estimates to service delivery efficiency and geographic access. |
|  | **Minor comments** |  |
| 29. | Clarity and readability: Some sentences are long and technical (especially abstract). Simplify for broader public health audience throughout: | Revised |
| 30. | E.g. Line 129: “but rather to estimate how different scales of sample testing expansion could affect overall program costs.” Rather “but rather to assess how varying levels of testing scale-up may impact overall programme costs.” | We thank the reviewer for this stylistic suggestion. We agree that the proposed wording is concise; however, we have retained the original phrasing as it more precisely reflects the analytical intent of the tool.  Specifically, the distinction between “different scales of sample testing expansion” and “varying levels of testing scale-up” is not merely stylistic in this context. The original wording was chosen to emphasize that the tool evaluates discrete scenario-based expansions in testing volume and configuration, rather than modelling a continuous or incremental functional relationship between scale and cost outcomes.  We have therefore kept the original sentence to preserve methodological precision and consistency with the scenario-based structure of the analysis. |
| 31. | First sentence of introduction is hard to follow | Revised as follow The World Health Organization (WHO)-recommended molecular rapid diagnostic tests (mWRDs) - a broad class of WHO-endorsed nucleic acid amplification tests (NAATs) capable of simultaneous TB detection and drug susceptibility testing (DST) — and these were used as the initial test in fewer than half of newly diagnosed TB cases in 2024[1]. The most widely deployed platforms in this class are the Xpert MTB/RIF Ultra (Cepheid) and Truenat (Molbio), both low-complexity automated NAATs (LCaNAATs). |
| 32. | Sentence on Line 66: Should the MiniDock MTB and Truenat MTB Ultima be included both as examples of NPOC (currently MiniDock MTB repeated mention and TrueNat only included at end of sentence). Later in Line 75 refer to the MiniDock MTB as “(e.g., MiniDock by Pluslife)” – be consistent. | The section is revised as follow: Thankfully, a new emerging class of simplified, portable molecular tests — near-point-of-care NAATs (NPOC-NAATs), hereafter referred to as NPOC platforms, (e.g., MiniDock MTB Test (MiniDock MTB))- have been developed for decentralized deployment at peripheral health facilities and community settings. Unlike LCaNAATs, NPOC platforms require minimal infrastructure, can accommodate non-sputum specimens such as tongue swabs, and are substantially lower in cost. Multicounty evaluations have shown that the NPOC achieves a sensitivity of 91.1% (95% CI: 82.1–95.9) and specificity exceeding 98% on sputum swabs [6], with WHO-pooled data across studies reporting a summary sensitivity of 85.4% (95% CI: 82.2–88.1) and specificity of 97.6% (95% CI: 96.9–98.1) on sputum. These estimates meet or approach the WHO Target Product Profile (TPP) thresholds of ≥90% sensitivity and ≥98% specificity for near-POC TB tests on sputum, and substantially outperform smear microscopy. Importantly, with sputum swab-based testing, sensitivity for NPOC differed by only −3.0% (95% CI: −8.6, 2.6) compared to sputum Xpert Ultra, with specificity exceeding 98% [6] — a margin that is not statistically significant and supports the clinical equivalence of the two platforms in the populations studied. |
| 33. | Consistency with abbreviations – e.g. near POC in Line 73 to NPOC. | updated |
| 34. | Tables: Large cost tables (e.g., Tables 3 & 4): Hard to interpret quickly. Suggest adding per test costs or cost per case detected. | We thank the reviewer for this helpful suggestion. We agree that metrics such as cost per test or cost per case detected can enhance interpretability in many contexts. However, we have not added these outputs in the current manuscript to remain consistent with the stated scope of the tool.  Specifically, the tool is designed as a high-level financial planning instrument that reports total programmatic costs under different scenarios, rather than a cost-effectiveness framework. Deriving metrics such as cost per case detected would require incorporating diagnostic performance parameters (e.g., sensitivity, specificity, and disease prevalence), which are intentionally outside the scope of the current model.  Additionally, per-test cost metrics are implicitly available within the tool through total costs and testing volumes, allowing users to derive these values if needed for their specific context.  To maintain clarity and avoid introducing partially specified effectiveness assumptions, we have retained the current presentation of total costs while clarifying this rationale in the manuscript. |
| 35. | Missing links: App and code are referenced but not clearly linked in manuscript. Ensure accessible URLs | the app is available here  https://saminap.shinyapps.io/minidock/  the codes are available here, due to size of the document, it is saved as a zip file that needs to be downloaded and open  https://zenodo.org/records/18653637  revised as follow: The resulting application script (app.R) was developed using the Shiny package and deployed on a cloud-based platform (Fig 1) to provide interactive, free access to users worldwide ([available here](https://saminap.shinyapps.io/minidock/)) (link: <https://saminap.shinyapps.io/minidock/>). The complete codebase is [accessible here](https://doi.org/10.5281/zenodo.18653637); link: https://zenodo.org/records/18653637. |
| 36. | Is the correct word not “microscopy sites” instead of “microscopic sites”? | Addressed, six typos were fixed throughout the document. |
| 37. | Very little justification or references provided for assumptions. | It is important to emphasize that this study was not designed as a formal economic analysis; rather, the scenario analysis serves to illustrate the functional outputs and operational utility of the diagnostic decision-support tool across varying programmatic conditions. As explicitly stated in the methods section, all assumptions were systematically informed by publicly available data and direct personal communications with experienced in-country program managers — the same rigorous approach employed to capture site-level mWRD and microscopy data and to construct hypothetical countries A and B, analogous to Bangladesh and Indonesia, respectively. |
| 38. | (e.g. line 212 assumed all tests start at 5.6m in year 1) | Revised as below:  For scenario 1, we used only the molecular test targets, and for scenario 2, we used the targets for all tests (including microscopic and molecular tests) from 2028 to 2030, as specified in Country A’s National Strategic Plan (NSP) for 2026-2030. |
| 39. | E.g. line 192: “We thought that when NPOC is rolled out, the number of samples needed to be transferred to LCaNAAT site for primary testing will go down to 5% in Scenario 1.” | Revised as below: Considering the portability of NPOC and a more decentralized scenario, it was hypothesized that the implementation of NPOC would result in a reduction of sample transfers to the LCaNAAT site for primary testing to approximately 5% of the current volume under Scenario 1 . T |
| 40. | This phrase undermines credibility and reads as circular and unscientific. “Additionally, storage and distribution costs were conservatively estimated at 2% based on our assumption.” Please check for more of these and word better, for example: “Additionally, storage and distribution costs were assumed to be 2% of baseline costs, based on simplifying assumptions in the absence of empirical data.” | Revised as below:  The model estimates the number of reflex tests needing transfer to LCaNAAT sites for drug susceptibility testing (DST) based on the country’s implied positivity rates. Notably, diagnostic commodities procured through the Global Fund are currently exempt from customs and import duties in Country A. Nevertheless, a 2% charge for shipping and handling was incorporated into this analysis to account for associated logistical costs. Furthermore, storage and distribution costs were conservatively estimated at 2% of commodity value, in the absence of empirical data, consistent with assumptions applied in similar health economic analyses. |
|  |  |  |

Comments from reviewer 2

|  | **Features of the tool** |  |
| --- | --- | --- |
| 41. | Can we call this tool as a cost estimation software for TB diagnosis? | Thank you for your suggestion, but this is not a software, rather we have used R software to develop this tool. |
| 42. | Whether this tool can provide cost estimates for different TB diagnostic algorithms? Since TB is not diagnosed by a single tool; it is a combination of tools following the diagnostic algorithm. | Agreed, we are focusing here only on the primary test done by mWRDs, no other tests were considered. |
| 43. | It is stated that the purpose of this tool is not to conduct cost-effectiveness analyses. However, it would be useful to clarify whether the tool can generate any cost-effectiveness outputs. If not, can it be considered suitable for cost-minimisation analysis. Otherwise, costing alone, when assessed independently, may not provide full economic evaluation. | The purpose of the tool is not to conduct economic analysis. We clearly state (lines 99–111) that the purpose of the tool is to describe and operationalize cost estimation, not to conduct formal cost-effectiveness or budget impact analyses. |
| 44. | Is it possible to add or modify the input parameters in the model or is it fixed? | Yes, all parameters are modifiable |
| 45. | Is there any uncertainty analysis included in this tool? To find out how robust are the results. | No, it is not an economic evaluation or costing analysis, therefore, no uncertainty analysis was conducted. |
| 46. | Whether a user manual or in-built guidance is available within the tool to support users in understanding and effectively utilizing its features | Yes, each step is described in the tool. |
| 47. | Whether the auto generated graphs are editable | No, but the results can be downloaded in excel and the graphs can be modified. |
| 48. | It would be useful to clarify whether the tool has an automated report generation feature. | Yes as mentioned in the tool description, the result can be downloaded in excel format |
|  |  |  |
|  | **Scenario analysis** |  |
| 49. | The authors have currently used two scenarios. It would be useful to explore whether additional scenarios could be included. If so, is there a maximum number of scenarios that the tool can accommodate. This can be added in the manuscript. | The tool is limited to two scenarios. However, the users can modify them according to their need. For example, they can adjust the microscope number, LCaNAAT number, test volumes etc. |
| 50. | When calculating cumulative costs over the years, whether discount or inflation is taken into consideration | Since this is not economic analysis, inflation or discount rate was not considered. |
| 51. | This hypothetical model appears to overestimate cost savings, reporting 56.4% in country-A for Scenario-1 based on current testing trend and 60.5% in country –B for Scenario-2 based on ambitious testing goals. This may require further justification or sensitivity analysis to support these estimates. | Unsure I have understood the comment clearly, there is no possibility of over estimation, rather it has underestimated some cost |
| 52. | It was mentioned that the results highlight the potential of cheaper molecular diagnostics to significantly improve access to mWRDs. It needs clarity. | It has been clarified in the discussion section. |
|  |  |  |
|  | **Other comments** |  |
| 53. | It is unclear whether the manuscript aims to highlight the innovation of the tool itself or the results generated by the tool. This lack of clarity appears to dilute the main focus of the study**.** | Thank you for your thoughtful and constructive feedback. We have revised the manuscript to clarify its scope and positioning. The primary objective of this work is to describe the development and functionality of the tool rather than to conduct a full economic evaluation or prescribe specific policy strategies.  The tool is intended as a practical, user-friendly calculator that enables stakeholders to estimate and compare the costs of TB diagnostic testing using LCaNAAT and NPOC approaches under varying testing volumes. It was developed in close consultation with two national TB programs and has been iteratively refined through testing in multiple low-resource settings. Feedback from these stakeholders has been incorporated to ensure its relevance and usability for policymakers.  We have also strengthened the manuscript to better emphasize that the tool serves an illustrative purpose—helping users explore potential cost implications and identify where savings may be realized by incorporating NPOC within broader diagnostic networks. Additionally, we have provided clearer justification of key assumptions and highlighted the tool’s limitations, particularly its focus on high-level cost comparisons rather than comprehensive economic or policy analysis. |
| 54. | Are there any limitations for the tool and scenario analysis? | Yes ,limitation section has been added in the discussion, While these findings provide encouraging evidence for the economic potential of NPOCs, several caveats should be acknowledged. The cost estimates are based on modeled assumptions and represent a simplified view of program expenditures. The analysis focuses on major cost categories—equipment, consumables, service contracts, and sample transport—while excluding indirect and micro-costs such as training, human resources, supervision, infrastructure depreciation, and waste management. These elements may differ significantly across settings and could influence total implementation costs. Additionally, pricing assumptions reflect ex-works values and may not capture variations in importation fees, supply chain inefficiencies, or local procurement practices.  While these findings provide encouraging evidence for the economic potential of NPOCs, several caveats should be acknowledged. The cost estimates are based on modeled assumptions and represent a simplified view of program expenditures. The analysis focuses on major cost categories—equipment, consumables, service contracts, and sample transport—while excluding indirect and micro-costs such as training, human resources, supervision, infrastructure depreciation, and waste management. These elements may differ significantly across settings and could influence total implementation costs. Additionally, pricing assumptions reflect ex-works values and may not capture variations in importation fees, supply chain inefficiencies, or local procurement practices. |
| 55. | Conclusion is not highlighting the tool | - Revised |
| 56. | Tables beginning with Table number 10. | - This is incorrect, it starts with 1 |
| 57. | All tables require professional editing to improve formatting. | - Revised |
| 58. | The first reference seems incomplete. | - Formatted according to the PLOS Global Public Health guideline |
