# Supplementary material for: An online, open-access, cost comparison tool for presumptive tuberculosis testing using low complexity, automated, nucleic acid amplification tests (LCaNAAT) versus near point-of-care (NPOCs) molecular tests
Source: PLOS Glob Public Health. 2026 Nov 4;6(11):e0006559. doi: 10.1371/journal.pgph.0006559 (PMC13734499; doi:10.1371/journal.pgph.0006559)
Supplement: Supplementary file 8 [file pgph.0006559.s008.docx]

August 11, 2026

Miquel Vall-llosera Camps
Staff Editor
PLOS Global Public Health

Dear Miquel Vall-llosera Camps,

Thank you for your careful review of our manuscript, “An online, open-access cost comparison tool for presumptive tuberculosis testing using low-complexity automated nucleic acid amplification tests (LCaNAAT) versus near point-of-care (NPOC) molecular tests”(PGPH-D-26-00841).

We have addressed the reviewer’s comments as outlined in the table below.

Please do not hesitate to let us know if any additional information or clarification is required. We would be happy to provide further details.

Thank you again for your time and consideration. We look forward to your response.

Yours sincerely,

Pushpita Samina

| Comments | Response |
| --- | --- |
| (1) adding a brief note in the Discussion on HR burden and operational feasibility; | The following sentence has been added to the manuscript “While NPOC may reduce per-test costs, higher testing volumes will increase staff workload and could create operational bottlenecks that limit the number of tests processed.” Line 432-434 |
| (2) providing better justification for the consumable cost reduction assumption; and | We have added the information source, it was discussed during the Global Fund’s NPOC implementation meeting. The meeting was attended by two co-authors. Please note that this is an “assumption” not a statement. |
| (3) clarifying the scenario customisation functionality in the tool. | Information added in the manuscript as below:  “For example, users wishing to model expansion of molecular testing to microscopy sites can enter the number of microscopy sites without existing molecular testing, while users in settings without existing LCaNAAT capacity can enter zero for the number of LCaNAAT modules. The tool then estimates the additional LCaNAAT capacity and NPOC requirements associated with the specified infrastructure and testing targets. This approach allows program managers to explore different levels of diagnostic scale-up and procurement using country-specific inputs without modifying the underlying model.” Line 162-168 |

Table : Response to reviewer’s comments
