# Supplementary material for: An online, open-access, cost comparison tool for presumptive tuberculosis testing using low complexity, automated, nucleic acid amplification tests (LCaNAAT) versus near point-of-care (NPOCs) molecular tests
Source: PLOS Glob Public Health. 2026 Nov 4;6(11):e0006559. doi: 10.1371/journal.pgph.0006559 (PMC13734499; doi:10.1371/journal.pgph.0006559)
Supplement: Peer review history

# Peer review: Decision Letter 0

**DOI:** 10.1371/journal.pgph.0006559.r001

**Authors:** Priya Rajendran

29 Mar 2026

PGPH-D-26-00841

An online, open-access, cost comparison tool for presumptive tuberculosis testing using low complexity, automated, nucleic acid amplification tests (LCaNAAT) versus near point-of-care (NPOCs) molecular tests

PLOS Global Public Health

Dear Dr Pushpita,

Thank you for submitting your manuscript to PLOS Global Public Health. After careful consideration, we feel that it has merit but does not fully meet PLOS Global Public Health’s publication criteria as it currently stands. Therefore, we invite you to submit a revised version of the manuscript that addresses the points raised during the review process.

Please submit your revised manuscript by April 30th 2026. If you will need more time than this to complete your revisions, please reply to this message or contact the journal office at globalpubhealth@plos.org. When you're ready to submit your revision, log on to https://www.editorialmanager.com/pgph/ and select the 'Submissions Needing Revision' folder to locate your manuscript file.

Please include the following items when submitting your revised manuscript:

- A letter that responds to each point raised by the editor and reviewer(s). You should upload this letter as a separate file labeled 'Response to Reviewers'.
- A marked-up copy of your manuscript that highlights changes made to the original version. You should upload this as a separate file labeled 'Revised Manuscript with Track Changes'.
- An unmarked version of your revised paper without tracked changes. You should upload this as a separate file labeled 'Manuscript'.

Guidelines for resubmitting your figure files are available below the reviewer comments at the end of this letter.

As the corresponding author, your ORCID iD is verified in the submission system and will appear in the published article. PLOS supports the use of ORCID, and we encourage all coauthors to register for an ORCID iD and use it as well. Please encourage your coauthors to verify their ORCID iD within the submission system before final acceptance, as unverified ORCID iDs will not appear in the published article. *Only*  the individual author can complete the verification step; PLOS staff *cannot*  verify ORCID iDs on behalf of authors.

We look forward to receiving your revised manuscript.

Kind regards,

Priya Rajendran, PhD

Academic Editor

PLOS Global Public Health

Journal Requirements:

1. Please note that PLOS Global Public Health has specific guidelines on code sharing for submissions in which author-generated code underpins the findings in the manuscript. In these cases, we expect all author-generated code to be made available without restrictions upon publication of the work. Please review our guidelines at https://journals.plos.org/globalpublichealth/s/materials-and-software-sharing#loc-sharing-code and ensure that your code is shared in a way that follows best practice and facilitates reproducibility and reuse.

2. Please amend your detailed Financial Disclosure statement. This is published with the article. It must therefore be completed in full sentences and contain the exact wording you wish to be published.

a. State the initials, alongside each funding source, of each author to receive each grant.

b. State what role the funders took in the study. If the funders had no role in your study, please state: “The funders had no role in study design, data collection and analysis, decision to publish, or preparation of the manuscript.”

c. If any authors received a salary from any of your funders, please state which authors and which funders.

3. Please provide separate figure files in .tif or .eps format.

For more information about figure files please see our guidelines:  LINK

https://journals.plos.org/globalpublichealth/s/figures

https://journals.plos.org/globalpublichealth/s/figures#loc-file-requirements

If the reviewer comments include a recommendation to cite specific previously published works, please review and evaluate these publications to determine whether they are relevant and should be cited. There is no requirement to cite these works unless the editor has indicated otherwise.

Reviewers' comments:

Reviewer's Responses to Questions

**Comments to the Author**

1. Does this manuscript meet PLOS Global Public Health’s publication criteria?

Reviewer #1: Partly

Reviewer #2: Yes

**********

2. Has the statistical analysis been performed appropriately and rigorously?

Reviewer #1: N/A

Reviewer #2: Yes

**********

3. Have the authors made all data underlying the findings in their manuscript fully available (please refer to the Data Availability Statement at the start of the manuscript PDF file)?

The PLOS Data policy

Reviewer #1: Yes

Reviewer #2: Yes

**********

4. Is the manuscript presented in an intelligible fashion and written in standard English?

Reviewer #1: Yes

Reviewer #2: Yes

**********

Reviewer #1: I have uploaded an attachment that details all my comments and concerns. See attached.

Reviewer #2: Manuscript number PGPH-D-26-00841

Title: An online, open-access, cost comparison tool for presumptive tuberculosis testing

using low complexity, automated, nucleic acid amplification tests (LCaNAAT) versus

near point-of-care (NPOCs) molecular tests

Overall comments: This manuscript focused on an important area of development of automated cost calculation software or tool (flexible cost calculator) especially relevant for health economics. I hope this tool will help decision makers understand the financial implications of expanding the newer diagnostic tools for TB detection with optimising resource use and accelerating TB case detection under the National TB Control Programme. They also provided results of two hypothetical scenarios for Bangladesh and Indonesia. Overall, this research addresses an important topic and well written manuscript, I recommend for publication subject to minor revisions. However, the following points should be emphasised.

Specific comments

Features of the tool

• Can we call this tool as a cost estimation software for TB diagnosis?

• Whether this tool can provide cost estimates for different TB diagnostic algorithms? Since TB is not diagnosed by a single tool; it is a combination of tools following the diagnostic algorithm.

• It is stated that the purpose of this tool is not to conduct cost-effectiveness analyses. However, it would be useful to clarify whether the tool can generate any cost-effectiveness outputs. If not, can it be considered suitable for cost-minimisation analysis. Otherwise, costing alone, when assessed independently, may not provide full economic evaluation.

• Is it possible to add or modify the input parameters in the model or is it fixed?

• Is there any uncertainty analysis included in this tool? To find out how robust are the results.

• Whether a user manual or in-built guidance is available within the tool to support users in understanding and effectively utilizing its features

• Whether the auto generated graphs are editable

• It would be useful to clarify whether the tool has an automated report generation feature.

Scenario analysis

• The authors have currently used two scenarios. It would be useful to explore whether additional scenarios could be included. If so, is there a maximum number of scenarios that the tool can accommodate. This can be added in the manuscript.

• When calculating cumulative costs over the years, whether discount or inflation is taken into consideration

• This hypothetical model appears to overestimate cost savings, reporting 56.4% in country-A for Scenario-1 based on current testing trend and 60.5% in country –B for Scenario-2 based on ambitious testing goals. This may require further justification or sensitivity analysis to support these estimates.

• It was mentioned that the results highlight the potential of cheaper molecular diagnostics to significantly improve access to mWRDs. It needs clarity.

Other comments

• It is unclear whether the manuscript aims to highlight the innovation of the tool itself or the results generated by the tool. This lack of clarity appears to dilute the main focus of the study.

• Are there any limitations for the tool and scenario analysis?

• Conclusion is not highlighting the tool

• Tables beginning with Table number 10.

• All tables require professional editing to improve formatting.

• The first reference seems incomplete.

Overall, authors made a good effort, but the current version needs a revision.

**********

what does this mean?). If published, this will include your full peer review and any attached files.

**Do you want your identity to be public for this peer review?**  If you choose “no”, your identity will remain anonymous but your review may still be made public.

For information about this choice, including consent withdrawal, please see our Privacy Policy

Reviewer #1: **Yes:**  Sarah Girdwood

Reviewer #2: No

**********

[NOTE: If reviewer comments were submitted as an attachment file, they will be attached to this email and accessible via the submission site. Please log into your account, locate the manuscript record, and check for the action link "View Attachments". If this link does not appear, there are no attachment files.]

Attachment Submitted filename: PGPH review.docx

Attachment Submitted filename: Online open access cost comparison tool.docx

# Author comment: Author response to Decision Letter 1

**DOI:** 10.1371/journal.pgph.0006559.r002

5 Jun 2026

Attachment Submitted filename: Response to reviewers.docx

# Peer review: Decision Letter 1

**DOI:** 10.1371/journal.pgph.0006559.r003

**Authors:** Miquel Vall-llosera Camps

14 Jul 2026

PGPH-D-26-00841R1

An online, open-access, cost comparison tool for presumptive tuberculosis testing using low complexity, automated, nucleic acid amplification tests (LCaNAAT) versus near point-of-care (NPOCs) molecular tests

PLOS Global Public Health

Dear Dr. Samina,

Thank you for submitting your manuscript to PLOS Global Public Health. After careful consideration, we feel that it has merit but does not fully meet PLOS Global Public Health’s publication criteria as it currently stands. Therefore, we invite you to submit a revised version of the manuscript that addresses the points raised during the review process.

Please submit your revised manuscript by Aug 12 2026 11:59PM. If you will need more time than this to complete your revisions, please reply to this message or contact the journal office at globalpubhealth@plos.org. When you're ready to submit your revision, log on to https://www.editorialmanager.com/pgph/ and select the 'Submissions Needing Revision' folder to locate your manuscript file.

Please include the following items when submitting your revised manuscript:

- A letter that responds to each point raised by the editor and reviewer(s). You should upload this letter as a separate file labeled 'Response to Reviewers'.
- A marked-up copy of your manuscript that highlights changes made to the original version. You should upload this as a separate file labeled 'Revised Manuscript with Track Changes'.
- An unmarked version of your revised paper without tracked changes. You should upload this as a separate file labeled 'Manuscript'.

Guidelines for resubmitting your figure files are available below the reviewer comments at the end of this letter.

As the corresponding author, your ORCID iD is verified in the submission system and will appear in the published article. PLOS supports the use of ORCID, and we encourage all coauthors to register for an ORCID iD and use it as well. Please encourage your coauthors to verify their ORCID iD within the submission system before final acceptance, as unverified ORCID iDs will not appear in the published article. *Only*  the individual author can complete the verification step; PLOS staff *cannot*  verify ORCID iDs on behalf of authors.

We look forward to receiving your revised manuscript.

Kind regards,

Miquel Vall-llosera Camps

Staff Editor

PLOS Global Public Health

**Journal Requirements:**

If the reviewer comments include a recommendation to cite specific previously published works, please review and evaluate these publications to determine whether they are relevant and should be cited. There is no requirement to cite these works unless the editor has indicated otherwise.

Please review your reference list to ensure that it is complete and correct. If you have cited papers that have been retracted, please include the rationale for doing so in the manuscript text, or remove these references and replace them with relevant current references. Any changes to the reference list should be mentioned in the rebuttal letter that accompanies your revised manuscript. If you need to cite a retracted article, indicate the article’s retracted status in the References list and also include a citation and full reference for the retraction notice.

[Note: HTML markup is below. Please do not edit.]

Reviewers' comments:

Reviewer's Responses to Questions

**Comments to the Author**

Reviewer #1: (No Response)

**********

publication criteria?

Reviewer #1: Partly

**********

3. Has the statistical analysis been performed appropriately and rigorously?

Reviewer #1: N/A

**********

4. Have the authors made all data underlying the findings in their manuscript fully available (please refer to the Data Availability Statement at the start of the manuscript PDF file)?

The PLOS Data policy

Reviewer #1: Yes

**********

5. Is the manuscript presented in an intelligible fashion and written in standard English?

Reviewer #1: Yes

**********

**Reviewer #1:**  I thank the authors for their detailed responses and am satisfied that the paper can move forward to publication. I do want to note a few concerns that I feel were not fully addressed in the revision, and which I think are worth the authors considering before final acceptance.

In general, the authors have relied on the tool’s intentional simplicity as a response to several of my comments. I understand and accept this framing, but a number of points deserved a more substantive engagement.

Human resources

I raised the need to discuss HR implications in the Discussion, and the authors indicated this was addressed but I cannot find the relevant text in the revised manuscript. This is worth including: while NPOC may reduce financial costs per test, higher testing volumes will increase staff workload and could create operational bottlenecks that limit the number of tests actually processed. Even a brief acknowledgement of this in the Discussion would strengthen the paper.

Scenario framing and customisation

The authors point to lines 211–236 as addressing my concern about Scenario 1, but I still think the most policy-relevant question is replacing smear microscopy with NPOC, rather than replacing existing LCaNAAT capacity. That being what it may, the authors note that the tool allows users to model this themselves, and I accept that this may be the case, but having tried using the Shiny application, I was unable to identify how to do so. There is no obvious way to change which platforms are substituted in each scenario, and the pre-set scenarios are not clearly described within the tool itself. I would ask the authors to add brief guidance notes within the application on how to customise scenarios.

Consumable cost reduction assumption

The assumption that NPOC consumable costs decline from USD 3.60 to USD 2.50 from Year 2 onwards is still not adequately justified. A footnote noting that costs decrease with volume is not sufficient, given how significantly this affects the modelled savings. The authors could either provide a reference to support this projection, or consider using USD 3.60 as a conservative baseline throughout.

Divisibility of LCaNAAT capacity

The authors acknowledged the limitation around proportional scaling of service and maintenance costs at the module level, but this is only briefly mentioned in the Discussion. Since real-world service contracts tend to be structured at the instrument level rather than the module level, this assumption may overstate savings and it is worth being a little more explicit about this in the Discussion.

To summarise, I am comfortable with the paper being published and recognise the value of a simplistic tool for high-level programmatic planning. I would ask the authors to consider, at minimum: (1) adding a brief note in the Discussion on HR burden and operational feasibility; (2) providing better justification for the consumable cost reduction assumption; and (3) clarifying the scenario customisation functionality in the tool .

**********

what does this mean?). If published, this will include your full peer review and any attached files.

**Do you want your identity to be public for this peer review?**  If you choose “no”, your identity will remain anonymous but your review may still be made public.

For information about this choice, including consent withdrawal, please see our Privacy Policy

Reviewer #1: **Yes:**  Sarah Girdwood

**********

[NOTE: If reviewer comments were submitted as an attachment file, they will be attached to this email and accessible via the submission site. Please log into your account, locate the manuscript record, and check for the action link "View Attachments". If this link does not appear, there are no attachment files.]

**Figure Resubmissions:**

# Author comment: Author response to Decision Letter 2

**DOI:** 10.1371/journal.pgph.0006559.r004

13 Aug 2026

Attachment Submitted filename: Response to reviwers 2.docx

# Peer review: Decision Letter 2

**DOI:** 10.1371/journal.pgph.0006559.r005

**Authors:** Julia Robinson

19 Oct 2026

An online, open-access, cost comparison tool for presumptive tuberculosis testing using low complexity, automated, nucleic acid amplification tests (LCaNAAT) versus near point-of-care (NPOCs) molecular tests

PGPH-D-26-00841R2

Dear Dr. Samina,

We are pleased to inform you that your manuscript 'An online, open-access, cost comparison tool for presumptive tuberculosis testing using low complexity, automated, nucleic acid amplification tests (LCaNAAT) versus near point-of-care (NPOCs) molecular tests' has been provisionally accepted for publication in PLOS Global Public Health.

Before your manuscript can be formally accepted you will need to complete some formatting changes, which you will receive in a follow up email. A member of our team will be in touch with a set of requests.

Please note that your manuscript will not be scheduled for publication until you have made the required changes, so a swift response is appreciated.

IMPORTANT: The editorial review process is now complete. PLOS will only permit corrections to spelling, formatting or significant scientific errors from this point onwards. Requests for major changes, or any which affect the scientific understanding of your work, will cause delays to the publication date of your manuscript.

If your institution or institutions have a press office, please notify them about your upcoming paper to help maximize its impact. If they'll be preparing press materials, please inform our press team as soon as possible -- no later than 48 hours after receiving the formal acceptance. Your manuscript will remain under strict press embargo until 2 pm Eastern Time on the date of publication. For more information, please contact globalpubhealth@plos.org.

Thank you again for supporting Open Access publishing; we are looking forward to publishing your work in PLOS Global Public Health.

Best regards,

Julia Robinson

Executive Editor

PLOS Global Public Health

***********************************************************

Reviewer Comments (if any, and for reference):

Reviewer's Responses to Questions

**Comments to the Author**

Reviewer #1: All comments have been addressed

**********

publication criteria?

Reviewer #1: Yes

**********

3. Has the statistical analysis been performed appropriately and rigorously?

Reviewer #1: N/A

**********

4. Have the authors made all data underlying the findings in their manuscript fully available (please refer to the Data Availability Statement at the start of the manuscript PDF file)?

The PLOS Data policy

Reviewer #1: Yes

**********

5. Is the manuscript presented in an intelligible fashion and written in standard English?

Reviewer #1: Yes

**********

Reviewer #1: (No Response)

**********

what does this mean?). If published, this will include your full peer review and any attached files.

**Do you want your identity to be public for this peer review?**  If you choose “no”, your identity will remain anonymous but your review may still be made public.

For information about this choice, including consent withdrawal, please see our Privacy Policy

Reviewer #1: No

**********
